# Tools of war and virtue–Institutional structures as a source of ethical deskilling

**DOI:** 10.3389/fdata.2022.1019293

**Published:** 2023-02-17

**Authors:** Sigurd N. Hovd

**Affiliations:** ^1^Peace Research Institute Oslo (PRIO), Oslo, Norway; ^2^University of Oslo, Oslo, Norway

**Keywords:** virtue ethics, extended cognition, AI, automation, skill, deskilling, institution

## Abstract

Shannon Vallor has raised the possibility of ethical deskilling as a potential pitfall as AI technology is increasingly being developed for and implemented in military institutions. Bringing the sociological concept of deskilling into the field of virtue ethics, she has questioned if military operators will be able to possess the ethical wherewithal to act as responsible moral agents as they find themselves increasingly removed from the battlefield, their actions ever more mediated by artificial intelligence. The risk, as Vallor sees it, is that if combatants were removed, they would be deprived of the opportunity to develop moral skills crucial for acting as virtuous individuals. This article constitutes a critique of this conception of ethical deskilling and an attempt at a reappraisal of the concept. I argue first that her treatment of moral skills and virtue, as it pertains to professional military ethics, treating military virtue as a sui generis form of ethical cognition, is both normatively problematic as well as implausible from a moral psychological view. I subsequently present an alternative account of ethical deskilling, based on an analysis of military virtues, as a species of moral virtues essentially mediated by institutional and technological structures. According to this view, then, professional virtue is a form of extended cognition, and professional roles and institutional structures are parts of what makes these virtues the virtues that they are, i.e., constitutive parts of the virtues in question. Based on this analysis, I argue that the most likely source of ethical deskilling caused by technological change is not how technology, AI, or otherwise, makes individuals unable to develop appropriate moral–psychological traits but rather how it changes the institution's capacities to act.

## Introduction

The prospect of autonomous artificially intelligent systems playing an increasingly prevalent part within the realm of warfare has been met by no small amount of concern from technologists, military ethicists, and the larger public alike. A central worry often expressed in this context has been the purported risk that the kind of automation these systems could facilitate and the risks of removing human ethical know-how from an area of paramount ethical concern. Former US Army Ranger Paul Scharre has provided, in his book *Army of None* (Scharre, 2018), a striking illustration of this conundrum from his own tours in Afghanistan in 2004. Here, Scharre describes working as a part of a sniper team sent to the Afghanistan–Pakistan border to scout Taliban infiltration routes. While doing so, they are spotted by nearby villagers, and not long after a young girl, “of maybe five or six” heads out their way with a couple of goats on the trail (2018, p. 3). Under the poorly constructed cover of herding goats, the girl is to serve as a spotter for Taliban fighters. As Scharre notes, in this situation this young child was legally classified as a combatant, and was thus, technically, also a lawful target under the laws of war. But to treat her as such was to the team unthinkable. On discussing what would turn out to be a failed mission in its aftermath, no one brought such a course of action up as an eventuality: “We all knew it would have been wrong without needing to say it. War does force awful and difficult choices on soldiers, but this wasn't one of them” (2018, p. 4).

The question this story raises for Scharre is what happens with the nature of warfare when human agents are increasingly fighting their wars through and with AI systems and agents? One may ask this question through a variety of lenses. One lens would be to ask whether an AI system could ever be expected to make similar kinds of decisions and what such a system would look like.^1^ Another lens is how the interaction with AI systems may come to change human beings' capacity for moral reasoning so that not even we can be expected to do so. That is, whether the implementation of and interaction with AI may come to change us in ethically problematic ways. From an ethical point of view, the latter of these lenses is arguably the most pressing. Not only does it address technology that is currently being implemented, but the question of whether an artificial moral agency is theoretically possible is a rather moot question if we are not capable of seeing the value in developing and implementing such systems. An influential concept through which this latter question has been raised is Shannon Vallor's concept of ethical deskilling (2015). As Vallor sees it, the introduction of AI technologies risks removing humans from the reality of war to a degree to which the skills constituting this moral wherewithal can no longer be developed.

While I think the notion of ethical deskilling to be a fruitful prism through which we can conceptualize some of the moral risks precipitated by this technological revolution, I will, in this article, argue we need to re-examine the notion of moral skill upon which this notion of deskilling is based. In so doing, I think there are good reasons to reject one central underlying assumption of Vallor, concerning both the nature and acquisition of these skills, as well as to rethink the most likely sources of ethical deskilling facing western militaries in the immediate future.

The article is divided into two main parts. In part one, I introduce Vallor's concept of moral skill and ethical deskilling and highlight some normatively problematic and moral psychologically implausible implications of a premise underlying this account. In part two, I go on to show how the concept can still be a highly relevant one if we take into consideration the institutionally mediated nature of professional military ethics.

## Part 1: Vallor's concept of military virtue and moral deskilling

### Moral skills: Some initial conceptual clarifications

Vallor's concept of ethical deskilling ties sociological literature on deskilling of workers within modern capitalism, originating in the work of Braverman, to neo-Aristotelian accounts of virtue represented by figures such as Hursthouse, McDowell, but most centrally, the study of Annas (McDowell, 1989; Annas, 1993; Hursthouse, 2002). Vallor's brief account of the concept of a moral skill, upon which this account of moral deskilling is supposed to rest, leaves, however, some ambiguity that will have to be addressed.

This ambiguity concerns how this notion of moral skill ties to the Aristotelian concept of moral virtue. Moral virtues are habitualized states of character disposing us to passion and action toward human flourishing. They are constituted as the mean between an excess and a deficiency (both vices), the way courage is the mean between cowardice and foolhardiness. The moral virtues move us to act by shaping our perception of the world (1109b). They allow us to recognize, in any given situation, what is good as good and what is bad as bad, good and bad here are understood in terms of human flourishing, and motivate us to realize the good (1113b). They are intrinsically tied to the intellectual virtue of practical wisdom, an intellectual virtue concerned with practical deliberation. Moral virtue provides us with the ends for which we act, and practical wisdom allows us to deliberate about the means through which they are enacted (1144a).

Vallor's concept of a moral skill isolates the epistemic capacities tied to the moral virtues, both from their motivating capacity as well as their role in deliberation:

Virtue must, therefore, be conceived as a habituated skill of discerning moral judgment joined with moral motivation and aim that guarantees the goodness of its use (Vallor, 2015, p. 110).

The way Vallor reads Aristotle, moral skills can be read as a stepping stone to proper virtue (Aristotle, 1984). They are, as she puts it:

[A] sort of scaffold or stable grafting site upon which virtue can (but may or may not) take hold; for genuine virtue is something more than moral skill or know-how, it is a state in which that know-how is reliably put into action when called for, and is done with the appropriate moral concern for the good (Vallor, 2015, p. 110).

Vallor's claim that virtue is somehow founded on a set of moral skills rests largely on Aristotle's assertion that the performance of a virtuous act requires: (1) knowledge; (2) the right motivation; and (3) that it proceeds from an unchangeable character (Vallor, 2015, p. 110; Aristotle, 1105a). From this claim, Vallor seems to conclude that a virtuous act is the conjunction of a set of discrete moral psychological phenomena where moral skills are a foundational part. But this analytic claim from Aristotle, about what virtuous acts require does not in and of itself say anything about the moral psychological functioning of a virtuous person. It does not substantiate a claim about the existence of a set of moral skills upon which moral virtues are founded.

Some ambiguity attaches, then, to Vallor's concept of a moral skill. In specifying its content, she leans heavily on the Aristotelian and neo-Aristotelian moral psychology, but the concept also introduces structures that are hard to find ground for in this literature and may even contradict it.

Given Vallor's cursory treatment of the concept, my pragmatic solution to this problem is to treat the concept as referring to the epistemic capacities associated with the Aristotelian concept of moral virtues but to refrain from following Vallor in treating it as a separate and foundational moral psychological phenomenon. This will allow me to avoid the thornier exegetic questions raised above, while also making it easier to tie her account of deskilling to the larger virtue ethical tradition, a tradition that after all plays a central role in her larger thinking on ethics and technology. From a pragmatic point of view, I see this move as justified by the fact that Vallor's argument for viewing the argument that AI technology may be a source of deskilling, if successful, would hold equally well whether we treat the concept of a moral skill to pick out the Aristotelian concept of moral virtue or as a more fundamental moral epistemological phenomenon. For, while Aristotle makes a clear distinction between virtues (*aretes*) and skills (*technai*), he does claim that they are analogous in being taught through habituation. This is also the central feature of moral skills upon which her argument about the risks of deskilling rests.

### Moral deskilling

The concept of deskilling originates from the sociological literature on work, in particular Braverman's *Labor and monopoly capital* (1974), and it refers to the process through which certain forms of skill and craftmanship can come to be made redundant and thus eliminated from work processes due to technological and managerially induced automation. Vallor's concept of moral deskilling refers to the elimination of moral skills from a given professional space through similar technological and managerial innovations. For moral skills to develop in an agent, they are dependent on factors such as exposure to models of the skill, basic motivation and cognitive and emotional resources, and a practical environment that allows sufficient opportunities for habituation. Within the military sphere, Vallor sees the processes of automation facilitated by AI technology as running the risk of hampering the habituation of moral skills among human military personnel by removing them from the realities of war.

There exists a rich institutional tradition of education of virtue in most modern professional armies, especially within the officer or other leadership cores […]. But as with all virtues they cannot be acquired in the classroom, or even in a simulator. Only in the actual practical context of war, where situations are neither stable nor well-defined and where success and failure have lifelong moral consequences, can words like “courage” and “discipline” be more than empty slogans or aspirational terms that cannot by themselves direct one to their achievement (Vallor, 2015, p. 114).

Vallor's worry, then, is that in a world where warfare increasingly is conducted by the means of technology that performs tasks that formerly only could be performed by humans, we will lose the moral wherewithal to use this technology in a virtuous fashion (Vallor, 2015, p. 115). Thus she questions, for example, whether human supervisors of a future army of lethal autonomous weapons could develop the right moral skills to provide any meaningful input to these weapon systems:

A supervisor, in order to be legitimate authority, must have more experience and practical wisdom than the supervisee: in this scenario, what wisdom will future humans “on the loop” be able to offer the machines in this regard? (Vallor, 2015, p. 115)

While there very well may be something immediately intuitive about the idea that this kind of technological change can come to affect our capacity for moral cognition, I think there are good reasons to question one underlying premise of Vallor's particular argument for the potential for moral deskilling. Vallor's argument relies on the premise that military virtue is a *suis generis* form of virtue. Hence, military virtues like courage, discipline, honor, and respect have nothing in common with their non-military counterparts.

To see this, let us for a moment treat the military virtues as merely a subspecies of their respective general kinds so that military and personal courage are two species of the same general kind of virtue, which is courage. Do we have the same reason to fear that human combatants, removed from the harsh realities of war are at risk of moral deskilling? I would argue, no. For if military virtue is not a *suis generis* kind of virtue, we must recognize that even a moderately virtuous person has a vast array of epistemic resources to come to recognize what is morally called upon them to do in a new situation. Their predicament is not to develop an entirely new set of character traits, but to learn how to apply their character to a new situation, i.e., to get an appropriate situational awareness of what is going on. For them to be able to do this, they might have to learn a new set of practical skills, but the fact that technological developments call on us to develop one set of practical skills rather than another does not mean that we are left incapable of recognizing right and wrong as a result.^2^ This would only be the case if the necessary moral wherewithal required to make judgments about warfare had to be grounded in a very particular set of unmediated experiences of war. What this amounts to is treating military virtue as a *suis generis* form of moral cognition and virtue. I think there are good reasons to, on closer scrutiny, reject this notion both on normative and moral psychological grounds.

The notion that military virtue is a *suis generis* virtue is both normatively problematic and relies on questionable moral psychology. Assuming a broadly democratic outlook it is normatively problematic because it makes it difficult to see how one could justify any civilian control and oversight over the military if the normative standard on which the institution is to be judged are standard civilians who have no independent epistemological access to. How is, for example, the US Congress in any different position in relation to its military than the human supervisors are in relation to the army of lethal autonomous weapons mentioned by Vallor above? Furthermore, the moral psychological account of such a view of military virtue would seem to imply that it is simply highly implausible. If the appropriate moral skills necessary to act virtuously in a military setting can only be acquired within the context of war, then the number of such experiences afforded the average modern soldier would seem to highly underdetermine the presence of any habituated moral skill. Even adding the time that modern military personnel are given to reflect on the moral obligations attached to their professional roles, be it the regular soldier or even members of the officer core, it is hard to see how anything resembling bonified habituated virtues could be developed solely on this basis. The training would have to build upon preexisting moral skills. But if military virtue is a *suis generis* virtue, how could such pre-existing moral skills play any such role?

To illustrate the problem at hand, I wish to consider a case Vallor points to as a paradigmatic instance of military virtue: the case of Hugh Clowers Thompson Jr. and his actions during the My Lai Massacre. On 16 March 1968, US Army soldiers committed the mass murder of between 347 and 504 unarmed people in two hamlets, My Lay and My Khe, of the Son My village in Vietnam. While the incident was and is a blight on the reputation US army, it has also come to be known as a case of incredible moral heroism through the actions of Warrant Officer Hugh Clowers Thompson. The village Son My was suspected by the US army of being a Viet Cong stronghold. Thompson and his observation helicopter crew were given the task of assisting in a search and destroying the mission. The intelligence was wrong. Upon entering the village, the army was met with no resistance and no sign of the enemy. All the same, they went on to indiscriminately execute its population, men, women, and children. Thompson, upon realizing that a massacre was taking place did everything in his power to stop it, going so far as to land the helicopter between fleeing civilians and advancing land troops (Angers, 2014).

To Vallor, Thompson's actions are a perfect example of the kind of moral wherewithal human beings are capable of, even in as extreme circumstances as found in war. I wholeheartedly agree. But are the moral skills constituting this wherewithal grounded solely or even predominantly in experiences of war? A closer look at Thompson's life reveals many factors which may have had an impact on his capacity to embody such a heroic response, beyond his experience as a military officer. As Trent Angers has noted in his biography of Thompson, this is a person with an upbringing that in many ways prepared him for this moment (2014). Thompson was raised in a family environment that valued discipline and integrity. He had been a boy scout and had been actively involved in the Episcopal church. While being raised in Georgia, his family denounced the racism and discrimination taking place in the south. If we were to take all these factors into account–his strong ties to a religious community, the moral examples present in his life, and his engagement in organizations putting a strong value on service–is it impossible to imagine that Thompson would be able to manifest the kind of moral traits of character necessary to supervise military action mediated by lethal autonomous weapons? If we can imagine this, we need to rethink our account of ethical deskilling and the understanding of moral skills upon which it rests.

## Part two: Moral skills and the social environment

### On moral deskilling: Broadening the term

It is worth taking a closer look at the sociological origins from where Vallor appropriates the concept of deskilling. When Braverman, in *Labor and Monopoly Capital*, described the introduction of scientific management theory (Taylorism) in the late nineteenth and early twentieth century as a process of deskilling, he is doing so with reference to labor as a social and political class (Braverman, 1974, p. 3, 294). The concept is closely tied to a Marxist understanding of capitalistic production and exploitation. Contrary to popular opinion, then as now, viewing western industrialized economies as tending toward a more and more skilled workforce, Braverman saw them as tending toward polarization. Capabilities earlier embodied in a class of craftsmen had been appropriated into the means of production. Management techniques, such as the division of production processes into ever smaller and more specialized labor tasks, lowered the skill required by any laborer, making each worker an ever more disposable part of the production process. The amount of knowledge put into the production process gets higher as industrialized societies are increasingly dependent on the skills of managers and engineers, but “the mass of workers gain nothing from the fact that the decline in their command of the labor process is more than compensated for by the increasing command on the part of managers and engineers” (Braverman, 1974, p. 294 ). To Braverman, then, his analysis of deskilling does not, first and foremost, refer to a loss of capabilities within a society or organization but to a change in the relation of power between labor and capital. To Braverman, deskilling refers to laborers' continuous loss of control over the production process *vis-à-vis* capital.

From a certain virtue ethical point of view, Braverman's account of deskilling makes for a compelling indictment of a capitalist economic system's capacity to provide a good foundation for human flourishing. In fact, it fits rather well with the anti-modern, neo-Aristotelian virtue ethics of Alisdair Macintyre (Macintyre, 2016, p. 91). If the notion of moral deskilling is to be applicable to the field of military professional ethics and professional ethics at large, however, some further translation work is needed. If we look at the types of organizations with a strong tradition for professional ethics, we are talking about institutions whose inner functioning cannot be captured in purely market economic terms. The purpose of these institutions, such as military, health and research institutions, judicial institutions, and so forth, is on a normative level to realize the common goods or values intrinsic to human flourishing. A military, for example, exists to safeguard the sovereignty of a state, maintaining its monopoly on violence within its territory by hindering interference from external threats. As such the legitimacy of its actions is tied up with the legitimacy of the state as a political entity. If a state's military conducts itself in a way that undermines the principles upon which the state's legitimacy rests, it is, at least on a normative level, undermining itself as an instrument of the state's political power.

Our theoretical interests in interrogating the possibility of ethical deskilling differ, therefore, in some crucial respects from the ones motivating the larger sociological literature on deskilling. Our worry that some crucial ethical wherewithal is being lost as ever-new facets of our active lives are being mediated by autonomous technology does not, first and foremost, pertain to the wellbeing of the professional, but the well-functioning of the institutions–to their ability to realize a common good. The reason we consider these skills inherently valuable has to do with the fact that the principles guiding the ethical wherewithal of these professionals are also constitutive of what it is that makes their institutions well-functioning. From this institutional perspective, it is therefore largely ethically unproblematic if ethical knowledge, which was prior embodied in the intuitive know-how of professionals, is now manifest in technological design and institutional structures. What is worrying is the risk of losing ethical knowledge on an institutional level.

To credibly substantiate this worry, we need an account of moral skills that: first, sheds light on the essential role played by the skill for the ethical functioning of the institution; and, second, how the introduction of a given technology may disrupt the functioning of this skill. In what follows, I will highlight a particular feature of the kind of ethical professional wherewithal theorized and cultivated within the field of professional military ethics that may help to provide the basis for such an account. I will argue that the kind of moral skills we want to see exercised by military professionals are essentially dependent on a larger institutional context, and this dependency makes them vulnerable to a particular kind of moral deskilling in the face of technological disruption.

### Skills, deskilling, and the social environment

As skills are always embodied in concrete individual persons who possess them as capabilities to act, it is perhaps natural to think of the phenomenon of deskilling through an individualistic psychological frame. On such a view, we will think of deskilling as a process through which an individual either fails to gain or lose a capability through either a lack of practice or a change in other abilities underpinning the skill. I may never become a good trumpetist because of a lack of disciplined training. I may once have been a decent trumpetist but let the skill atrophy. Or, alternatively, I may no longer be a great trumpetist due to an injury to my lung. But skills, as capabilities to act, are arguably almost always environmentally embedded phenomena. In describing what I am capable of doing, I am usually also making some reference, however implicit, to an environment facilitating and sometimes also partly constituting these capabilities. In analyzing the activity of tying one's shoelaces, one must make some kind of reference to the laces being tied, and thus, this environmental feature can be said to be a constitutive part of the skill of shoe tying. The environmental features that can facilitate or constitute a skill are not restricted to the physical environment. Professionals such as stock traders, auctioneers, and rhetoricians possess skills facilitated by the social environment in which they were developed, skills that may rely on features of this social environment to a degree to which they can be said to partly constitute what it means to perform them all.

If we take these forms of environmental dependency into account, new possible forms of deskilling also come to the fore. As these skills usually are dependent also on environmental variables, changing these variables can change the individual's capabilities. At a height of 100 m above sea level, I may be a decent trumpeter, but less so in the low air pressure of high altitudes. I may be a skilled rhetorician under certain social circumstances but not under others. In this way, changes in the physical and social environment in which an agent is embedded may either enhance or diminish the skillfulness with which they are able to face a given situation.

The skills of professional athletes are an interesting example in this regard. To be a skilled professional basketball player, cross-country skier, or runner involves interacting with an environment that is socially and technologically mediated, where what it means to be a skilled athlete, in any of these sports, is in part determined by its social and technological features. In defining standards of excellence within the game of basketball, for example, we must make some reference, either implicitly or explicitly to the rules of the game. Throughout the history of the game, these rules have changed. Many of these changes, instituted by leagues such as the NBA or the NCAA, have been made in order to either enhance or diminish certain star players playing within the league at the time. To mention a few, in 1947, the NBA banned zone defense in order to enhance the impact of dominant players like Neil Johnston, Dolph Schayes, and Bob Petit (Warond, 2017); in 1951 and 1964, the league expanded the lane, first from 6 to 12 feet and then from 12 to 16, encouraging guard and wing play and curbing the power of taller star players such as George Mikan and Wilt Chamberlain. From 1967 to 1976, the NCAA banned dunking, responding allegedly to the strength of Lew Alcindor (Caponi, 1991, p. 4); and in 2001, the NBA reinstated the zone defense to offset the pure physical dominance displayed by Shaquille O'Neil (Warond, 2017).

Such changes in the conventional norms governing the game may, from the perspective of the individual player, be described as an instance of deskilling. A player may have developed skills throughout their career, the significance of which may be greatly diminished as a result of the changing rules. Physical attributes which at one point gave them a crucial advantage may no longer be as significant, and new skills and physical attributes may come to the fore as essential to the game. It may also be described as an instance of deskilling from the perspective of the team. Basketball is a team sport, and the significance of any particular skill in making a good basketball player is determined by its role in the collective effort of winning the game. In light of a significant change in the rules of the game, roles and tactics may become obsolete, and a team, once pre-eminent, may find their style of play an ill-fit in this new environment.

Within sports, as in so many other instances of expertise, this kind of deskilling through environmental change is often precipitated by technological development. In cross-country skiing, for example, changes in ski design and vaxing technology have completely changed the physical profile of elite professional skiers, now increasingly favoring athletes with a higher muscle mass. If a professional skier from the 1970's was transported to the world championship, anno 2022, they would consequently suffer from deskilling purely because of changes in the technological environment they are interacting with. For the Nordic countries, who for cultural and historic reasons have invested heavily both in skiing and vaxing technology as well as empirical research into professional development, these changes in the technological environment have given them a comparative advantage, leaving other once strong skiing nations in the dust.

### Military ethics and institutional environments

If we are to look for potential threats of moral deskilling within the military as a result of technological disruption, I would propose that the most pressing threats are analogous to the ones presented above; not precipitated by changes in the moral character of military personnel seen in isolation, but by changes in their environment, in particular, the institutionally mediated environment in which military personnel are embedded. To defend this view, I will in this section provide a brief account of the role of institutional mediation in military ethics, and in the next section provide an example of how this institutional context can be a source of deskilling.

This notion of an institutionally mediated environment calls for some further conceptual clarification. By an institution, I mean to refer to an organization constituted by an embodied structure of differentiated roles (Miller, 2010). These roles are defined in terms of a set of interdependent tasks with reference to a shared overarching end, a set of formal rules regulating suitable behavior, as well as a set of informal cultural norms. Institutional behavior, like all human behavior, is also supported by a set of material and technological conditions. In talking about an institutionally mediated environment, I mean to refer to the space of action of agents embodying a given role within an institution, for example, how the institution through rules, cultural norms, and material and technological resources both constrains and extends the agent's own capabilities to act. Institutions are tools for collective action, they enable individuals acting through them to perform feats far beyond the power of any singular person. With this power comes moral obligations and social expectations which constrain how this role may be wielded in a morally and socially legitimate way.

The account of deskilling I wish to put forward assumes that moral skills associated with the military profession–skills usually described as military or martial virtues–are species of their more general kinds rather than a sui generis form of moral traits. Military courage is simply a specific form of courage, military loyalty is a specific form of loyalty, and military prudence is a specific form of prudence. Additionally, following a loosely Thomistic account of military virtue, I would argue that what characterizes these virtues as virtues of a specific kind is that they are exercised in the pursual of a common good–a precondition of human flourishing–that cannot be realized by any one private individual, i.e., securing the safety and autonomy of a polity against external threats.

Similar to Aristotle, St. Thomas saw the moral virtues, i.e., dispositions toward the good, as virtues proper, only in so far as they were guided by the intellectual virtue of practical wisdom, or prudence–excellence in deliberation practical deliberation for the sake of the good (1140a; ST I-II q47 a 2). One might be disposed to act in ways that happen to be courageous or temperate by means of a good upbringing or natural dispositions. However, unless oneself am capable of deliberating about the ends for which my nature and upbringing disposes of them, one is not truly courageous or temperate (1144b14-18; ST I-II q 65 a 1). Through virtue, I recognize and am inclined toward the good and through practical wisdom, I deliberate about means to realize it, specifying its content in this particular case. But the good toward which virtue directs us and our practical wisdom deliberates about in military affairs is one that can be realized solely by our own actions. It must be pursued by the polity collectively. Military prudence, then, because it is ultimately concerned with the safety and autonomy of the polity, a good that no single member of the polity has the power to realize by themselves, may only be exercised if an agent's practical deliberation is integrated into a larger collective project. The institution of the military is a tool enabling the integration of individual actions into such a larger project. Additionally, military prudence is a virtue concerned with practical deliberation regarding collective action, as mediated by this institution, for sake of the common good.

As argued by Reichberg (2017), St. Thomas was thus also well aware of the essential role played by institutional mediation in the exercise of prudence within the military sphere. We see this reflected both in his understanding of political and military authority as being legitimized by the need for a coordinated endeavor to be guided by a unified will (ST II-II q 40a1), as well as in his treatment of the kinds of prudence required by humans engaged in acts of war.

St. Thomas' conception of military prudence is closely tied to his conception of prudent governance more broadly, as well as his understanding of the nature of legitimate political authority. Working from the premise that there are common goods that only can be realized through coordinated collective action, he consequently emphasizes a distinction between the prudence required of a ruler (*prudentia regnitiva*) and the prudence required of the ruled (*prudentia politica*), as well as recognizing a distinct form of *prudentia regnitiva* pertaining to military affairs (STII-II q 50 a4). For rational agents engaged in such collective endeavors, there exist, according to Aquinas, different standards of deliberative excellence, determined by the agent's role in the collective endeavor: a virtue of command and a virtue of obedience (Aquinas, 1981). But as Reichberg notes, obedience, as a virtue, is never blind. By treating both a commander's giving of orders and a subordinate's implementation of them as instances of deliberation and action falling under the confines of the intellectual virtue of prudence, they are both realized only insofar as they are guided by the common good:

[T]o be humanely exercised civic obedience requires a special part mode of deliberation; in this respect, purely personal prudence is an incomplete guide, for, in addition to reflecting on the implications for my private good, I must weigh the concordance of the command with the common good of the polity of which I am a member (Reichberg, 2017, p. 140).

Both the commander's and the soldiers' actions, then, are constitutive parts of a unified collective act that must be guided by the common good, as far as they are to be considered moral. For their actions to be guided by the common good means for both to be correctly attuned to the institution of which they are a part, an institution that mediates their actions through a set of differentiated roles.

We commonly do not think of institutional structures as being an essential part of moral reasoning. When, for example, Kant, in the Critique of Practical Reason (Kant, 1997, p. 118/5, 148), speaks of the moral law within, he is describing a fairly widely held picture of moral conscience and cognition. That is, as a process taking place in the deepest privacy of our own minds, our humanity, pure and simple, stripped of anything accidental, like sentiment or convention. From within such an internalist picture, it is perhaps natural to say that the best we may ask of our institutions is to not stand in the way of our conscience. Indeed, Hannah Arendt has shown us how institutional structures can come to be deeply detrimental to our capacity to recognize the call of morality, describing in her study of Adolf Eichmann, how legal and bureaucratic structures can facilitate acts of evil whose terribleness is matched only by their banality (Arendt, 1963, p. 72, 118). Yet, what St. Thomas's accounts of the ethics of war so perfectly illustrates is that our moral conscience does not always appeal to our humanity simpliciter. Sometimes its appeal is an appeal to us in virtue of our profession, as a nurse, a journalist, a judge, or a soldier.^3^ These roles are partly defined through the institutional structures into which they are integrated. Structures play a constitutive role in determining what ethical action means within a professional space because they determine what it means to embody a given professional role–invested by a society with unique powers and ethical obligations.

A useful prism to understand the role played here by these institutional structures is the concept of extended cognition. In their seminal article from 1998, *The Extended Mind*, Andy Clark and David Chalmers suggested that features of our external environment may not only facilitate processes such as calculation, memory, belief formation, orientation, and reasoning but may as well come to serve constitutive parts of these processes themselves. Cognitive tools, such as notebooks, cellphones, and abacuses, can thus be said to extend our mind beyond our body and into the environment itself (Clark and Chalmers, 1998). As was already argued then, there is no principled reason to think that parts of our social environment cannot play a similar function:

Could my mental states be partly constituted by the states of other thinkers? We see no reason why not, in principle. In an unusually interdependent couple, it is entirely possible that one partner's beliefs will play the same sort of role for the other as the notebook plays […] (Clark and Chalmers, 1998, p. 17).

More recently Shaun Gallagher and Anthony Crisafi have pointed to institutional structures, the ones constituting the institution of the law, as an instance of socially extended cognition in some ways even more radical than what Clark and Chalmers immediately had in mind:

There may be external resources that can carry out cognitive processes that in principle may not be possible to do in our head, and that we would have a hard time conceptualizing as something we could even refer to the phrase “if it were done in the head” (Gallagher and Crisafi, 2009, p. 47)

The law, which may be the product of previous generations, but is currently organized in legal institutions, operates like a mechanism that helps to accomplish our thought (Gallagher and Crisafi, 2009, p. 48).

The work of Clark, Chalmers, Gallagher, and Crisafi may suggest a promising perspective to think of the nature of the so-called martial virtues, as concerned with the attunement of our moral character to a unique cognitive tool: the military institution. Of course, much work remains in exploring the ultimate viability of applying this conceptual framework to the topic of professional military ethics–work that would take us too far afield from the aims of this present paper.^4^ What immediately can be achieved by taking up such a perspective, however, is to put the collective technologically mediated nature of military action in focus. Thus, if nothing else, it can function as a corrective to a moral psychological point of view still dominating most contemporary moral philosophy. Seeing professional military ethics through the prism of extended cognition invites us to the words of Clark, “to cease to unreflectively privilege the inner, the biological, and the neural” (Clark, 2011, p. 218), and as I hope to show in the next section, it can shed light on how our capacities for moral reasoning can be undermined by technological change.

### Military ethics and moral deskilling

Already in the short to medium term, the technologies falling under the rubric of AI holds the promise of issuing an era of unprecedented automation. So too in the military sphere, different levels of autonomy have already been introduced in a vast array of weapon systems and continue to do so in the future, opening new tactical and strategic advantages whose ramifications we are only beginning to understand. With the introduction of this technology comes also new challenges. One challenge I here wish to bring to the fore pertains to the scalable nature of autonomous weapon systems. Stuart Russel, who otherwise has expressed skepticism about the moral panic about lethal autonomous weapons, has nevertheless expressed some worry about just this feature:

[A] process is scalable if you can do a million times more of it with buying a million times more hardware. Thus Google handles a roughly five billion search requests per day by having not million of employees but million of computers. With autonomous weapons you can do a million times more killing by buying a million times more weapons precisely because the weapons are autonomous. Unlike remotely piloted drones or AK-47s, they don't need individual human supervision to do their work (Russel, 2019, p. 112).

If we were to borrow some quasi-Marxian economic terms, we could say, then, that AI holds within it the potential of transforming vast amounts of the human labor involved in the process of military action into pure “means of destruction” through the process of automation. This obviously constitutes a potentially massive expansion of the destructive potential of modern militaries; first of all, because labor is a much scarcer resource than capital; second, because the loss of one's own soldiers, is one of the biggest political restrictions for a polity to revert to force. I would argue that it is also a potential source of ethical deskilling.

The scalable nature of autonomous weapons means their introduction into a military will entail a massive expansion of the potential scale of the institution's actions. Seen in isolation, the actions it affords the institution might not seem qualitatively different from those already in its arsenal. But increasing the scale on which an institution may act can come to change the nature of these actions themselves. It is not given that individual humans operating within the institution will possess sufficient conceptual frameworks to apprehend the ethical implications of this increase in scale.^5^ The introduction of AI technology into military institutions may thus cause ethical deskilling among military professionals by changing the institution within which they act. They might possess the exact same personality traits, physical capabilities, and self-understanding as they did prior to the introduction of this technology. But just like the way a change in the rules of basketball can come to change what it means to be a good basketball player, a technological change might change the traits, capabilities, and self-understanding needed to attune oneself to the institution of the military in an ethical fashion.

Arguably, a recent example of this kind of technologically and institutionally grounded ethical deskilling is found in the use of unmanned aerial vehicles (UAVs or drones) as a part of the US government's war on terror. While Russel is right in highlighting the unique threat represented by the scalability of autonomous weapon systems, when seen from a more sociological perspective, what drone warfare represents to modern militaries bears strong analogies with the benefits represented by the scalability of autonomous weapons. Drones increase a state's air power by lowering demands put on the personnel operating the aircraft, both in terms of the training needed for a human to be able to man it and the risks subsequently placed on this asset (drone pilots are not in immediate risk of being shot down). Drone warfare has thus radically lowered the cost of both applying and projecting air power, financially, manpower-wise, and politically. As was born out in the US war on terror, this lowering of the cost of action enables an increase in scale.

Actions once increased in scale can come to undergo metamorphoses, not always predictable to those putting them into being. So we arguably saw this reflected in the Obama administration's legal and moral justification for the use of UAVs to take out members of Taliban and Al-Qaeda leadership. When the US State department's Legal Advisor Harold Koh first presented this justification, he argued that there is nothing about this technology, *per se*, that would make it contrary to the laws of war. Nor is there anything unprecedented about the way this technology had been used by the US armed forces, as the US has used airstrikes to take out enemy leadership going back to the second world war. In fact, they argued, the targeted nature of such strikes enables the US to wage this war in a way that is both limited and proportional. One obvious objection here is that to consider the technology *per se* is to consider an abstraction. It does not allow us to come to terms with the question of proportionality because it does not allow us to perceive scale. It does not allow us to see the cost for a civilian populace living under the watchful gaze of tools of death and destruction, loitering in the sky above them. It does not invite the question of whether in the name of a war on terror one would be justified in placing these people under a *de facto* reign of terror.

By an increase in the scale of the action in question, then, the moral obligations associated with the action can change as well. And there is no guarantee that any one operator supervising these weapon systems has the sufficient overview to catch such changes, as well as the normative frameworks to problematize them. The same goes for the decision apparatus that exists within the institution to regulate and supervise their actions and civilian institutions in charge of providing external oversight.

Consequently, then, when worrying whether UAVs and LAWs may precipitate a loss of capabilities for moral reasoning, it may be natural to formulate one's worries as a question of whether drone operators or artificially intelligent autonomous systems possess sufficiently similar qualities to human beings now performing similar actions. But as war is an intrinsically collective endeavor, professional military ethics cannot be concerned solely with individual actions, abstracted from the larger collective endeavors of which it is a part. The question of military deskilling must therefore be treated as an institutional, rather than a purely individual concern. The question we must ask ourselves is whether the decision structures governing these intrinsically collective actions will possess the right kind of knowledge, an incentive structures to see and respond to a changing moral landscape. Seen from this perspective, the more likely candidates for ethical deskilling may not first and foremost be found among the average operators, but within military leadership. It is here, after all, that responsibility for these collective actions as a whole resides.

## Conclusion

Vallor's notion of ethical deskilling is a powerful one, capturing a deep-seated ambiguity about technological change and autonomous systems within the military sphere. I have in this article looked closer at the seams of the conception of a military virtue underpinning Vallor's application of this concept: whether military virtue is a *suis generis* form of virtue. I have argued that we have reasons to reject it both on normative and moral psychological grounds. From a normative perspective, this view of military virtue seems to imply a kind of moral exceptionalism on behalf of military institutions within their domain from where it would be hard to justify any kind of civilian oversight or control. From a moral psychological point of view, it is simply implausible, as the experience of combat afforded the average soldier woefully underdetermines the emergence of any independent moral skill. Military virtue must in some way be grounded in virtue simpliciter if it is reasonable for us to expect it to develop at all.

I have subsequently offered an alternative account of ethical deskilling on the basis of a view of military virtue and professional military ethics as a particular form of extended cognition. On this account, what makes military virtue a specific kind of virtue is its mediation through the military institution. Military virtue is virtue exercised on behalf of a common good that can only be realized through coordinated collective action. The military as an institution exists to coordinate this collective effort and functions as a cognitive tool through which individuals can partake in it. I have proposed that the perhaps most plausible sources of ethical deskilling are grounded in just this institutional-embedded nature of military virtue. Technology changes the capabilities of institutions in ways that may neither be entirely predictable to those introducing it nor to people set to use it. Radical technological change such as the one promised with the advent of ever new and more complicated AI technology can, thus, become a source of ethics by disorienting the institution to the ethical implications of their collective effort.

## Data availability statement

The original contributions presented in the study are included in the article/supplementary material, further inquiries can be directed to the corresponding author.

## Author contributions

The paper was written solely by SH.

## Footnotes

^1^Asimov's laws of robotics is an attempt to answer this question from within the civilian sphere (Asimov, 1977). Stuart Russel's proposal for a design of a benevolent AI, and Bostrom and Yudkowsky's suggestion of designing such systems around decision trees, rather than neural networks, are yet others (Bostrom and Yudkowsky, 2011; Russel, 2019). The question of such designs for ethical AI could also be realized within the military space is an interesting ethical question, but as is about to become clear, it is not a central concern for this paper.

^2^Even if military virtue only could be developed on the basis of moral skills of the kind we saw sketched out by Vallor above, and these skills could only be developed on the basis of a very specific set of experiences of war, in so far as these moral skills provide a foundation for military virtue, this would again imply that military virtue is a *suis generis* kind of virtue.

^3^That social roles might be ethically informative is in one sense clearly recognized. Role ethicists such as Baril, Garcia, and Scheffler have all highlighted ways in which social roles can come to guide moral behavior (Garcia, 1986; Scheffler, 1997; Baril, 2016). Recently, Joseph Chapa has also argued, I think concvincingly, that this is form of ethics may shed an interesting light on the nature of the martial virtues, and the two often seemingly conflicting normative demands placed on the soldier, that practical efficiency and moral integrity (Chapa, 2018).

^4^Treating a mode of moral cognition as dependent on features in the environment that is historically contingent, brings up meta-ethical challenges that such a view will have to address. Perhaps the most pressing question one will have to answer is how it is that we expect such a cognitive process to generate judgements with the kind of universal force we commonly associate with moral judgements.

^5^Of course, this is only one of many ways in which the introduction of AI technology may come to change the institutional environment through which we act.
